# Computational study on novel natural compound inhibitor targeting IDH1_R132H

**DOI:** 10.18632/aging.204162

**Published:** 2022-07-07

**Authors:** Baolin Zhou, Fang Yang, Lei Qin, Jun Kuai, Lu Yang, Lanfang Zhang, Peisheng Sun, Guangpeng Li, Xinhui Wang

**Affiliations:** 1 Department of Oncology, First People's Hospital of Xinxiang, Xin Xiang 453100, China; 2 Department of Gastroenterology, The First Affiliated Hospital of Xinxiang Medical College, Xin Xiang 453100, China; 3 Department of Gastrointestinal Surgery, The First Affiliated Hospital of Xinxiang Medical College, Xin Xiang 453100, China; 4 Department of Emergency, The First Affiliated Hospital of Xinxiang Medical College, Xin Xiang 453100, China

**Keywords:** IDH1_R132H, cholangiocarcinoma, computational study, natural compounds

## Abstract

Isocitrate dehydrogenases (IDH) catalyze the oxidative decarboxylation of isocitrate to 2-oxoglutarate. IDH1 mutation has been reported in various tumors especially Cholangiocarcinoma, while the IDH1_R132H is reported to be the most common mutation of IDH1. IDH1_R132H inhibitors are effective anti-cancer drugs and have shown significant therapeutic effects in clinical. In this study, two novel natural compounds were identified to combine respectively with IDH1_R132H with a stronger binding force with conductive to interaction energy. They also showed low toxicity potential. Molecular dynamics simulation analysis demonstrated that the candidate ligands-IDH1_R132H complexes is stable in natural circumstances with favorable potential energy. Thus, Styraxlignolide F and Tremulacin were screened as promising IDH1_R132H inhibitors. We provide a solid foundation for the design and development of IDH1_R132H targeted drugs.

## INTRODUCTION

Cholangiocarcinoma is an invasive adenocarcinoma, and origins from the malignant growth of the biliary duct epithelium [1]. Depending on their anatomic location, cholangiocarcinoma is generally categorized as intrahepatic or extrahepatic [2]. According to the American Cancer Society, the five-year survival rates of noninvasive intrahepatic cholangiocarcinoma and extrahepatic cholangiocarcinoma were 15% and 30%, respectively, whilst the 5-year survival rates of the two metastatic subtypes have significantly reduced to 2% [3]. Currently, the only curable therapy for this disease is surgical resection, which might include liver transplantation in some extreme or severe cases. After surgery, the beneficial adjuvant treatment is radiotherapy combined with chemotherapy [4]. Although chemotherapy is an important part of cholangiocarcinoma’s comprehensive treatment, the tolerance of tumors to chemotherapy has blocked its development [5, 6].

Isocitrate dehydrogenase 1 (IDH1) mutation is reported as a gain-of-function mutation in up to 25% of cholangiocarcinoma, especially intrahepatic cholangiocarcinoma [7, 8]. A relevant study shows that IDH1 and IDH2 mutations predominate in cholangiocarcinoma, while IDH2 contributes only partially (2–6%) [9]. Yuchen Jiao, et al. raised that cholangiocarcinoma patients with IDH1 mutation compared to IDH1 wildtype patients had 3-year overall survival significantly reduced [10], which exposes that IDH1 mutations are key points in cholangiocarcinoma genesis.

IDH1 mutates in cholangiocarcinoma gain neomorphic enzymatic activity, whereby IDH1 is an important metabolic enzyme, and IDH1 can converts the NADPH-dependent reduction of α-KG to 2-hydroxyglutarate (2-HG) [11, 12]. The accumulation of 2-HG leads to cellular changes in overloaded cellular metabolism, redox status, epigenetic regulation, and DNA repair, which are related to histones and typical CpG island hypermethylation phenotypes [13–15]. Taken together, these studies suggest that tumor growth may have acquired additional mutations that allow them to increase their proliferative capacity [16]. In addition, the binding site in arginine 132 (R132) is the most common mutation of IDH1 in cancer [17]. The most frequent IDH1 mutation hotspot is in codon 132 (IDH1-R132H mutation) [18]. If applied early in the treatment of gliomas, IDH1 inhibitors can prevent the spread of the cancer and decrease the potential side effects of radio- and chemo- therapy [19].

Therefore, identifying effective leading compounds which are capable of inhibiting IDH1-R132H mutations is promising in drug improvement and cholangiocarcinoma treatment. Currently, several inhibitors of IDH1-R132H have been founded, including Vorasidenib and Ivosidenib, which are the most developed inhibitors [20–22]. However, the negative effect of IDH1-R132H on drug resistance in cancer chemotherapy has been reported in several recent publications [23, 24]. Therefore, more efficacious IDH1-R132H targeted drugs were needed urgently. In this research, we employed variety of biological and chemical structure methods to screen leading compounds with inhibitory effects to IDH1_R132H. To provide basic groundwork for the improvement and study of IDH1-R132H inhibitors, this research established a series of candidate drugs and their pharmacokinetic characteristics.

## RESULTS

### Virtual screening of compounds against IDH1-I23H

**Figure 1:**
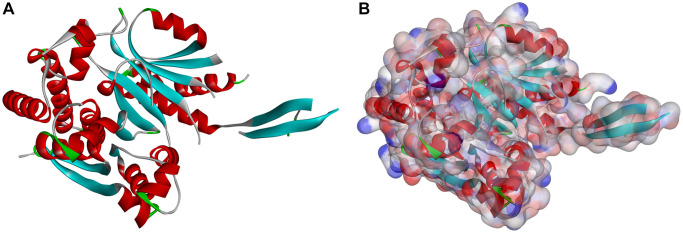
**Molecular structure of IDH1_R132H.** (**A**) Initial molecular structure. (**B**) Surface of binding area added. Blue represents positive charge, and red represents negative charge.

The ligand-binding pocket of IDH1_R132H was selected as a reference site as a noteworthy regulating site. The small molecules bound to the active site Cys145 can prevent a damaged alkyl group on DNA from docking at IDH1_R132H, which blocks the DNA repair of damaged cells. Figure 1 shows IDH1_R132H’s molecule structure. A sum of 17,931 bio-sourced named product molecules available for sale were chosen from the ZINC15 database. We chose an inhibitor called Vorasidenib and Ivosidenib as the reference compounds. After screening, there are 8763 compounds can bind to IDH1_R132H. The top20 compounds with higher LibDock scores showed in Table 1.

**Table 1:** Top 20 ranked compounds with LibDock scores.

**Number**	**Compounds**	**Trivial name**	**Libdock score**
1	ZINC000004098643	Epsilon-Viniferin	147.3
2	ZINC000042851784	Homoaromoline	144.166
3	ZINC000049872065	Andropanoside	139.69
4	ZINC000002033589	Karsil	138.261
5	ZINC000038148193	Fangchinoline	136.916
6	ZINC000030726940	Obaberine	136.439
7	ZINC000028539727	Dehydroxynocardamine	136.2
8	ZINC000021992902	Neoandrographolide	135.574
9	ZINC000049872393	Styraxlignolide F	135.121
10	ZINC000030729923	(9-Cis,9’-Cis)-7,7’,8,8’-Tetrahydro-Y,y-Carotene	134.363
11	ZINC000008220036	2-Hexaprenyl-3-Methyl-6-Methoxy-1,4 Benzoquinone	134.014
12	ZINC000030726863	Cepharanthine	133.907
13	ZINC000014727602	5,7,3’,4’-Tetrahydroxy-6,5’-Diprenylisoflavone	132.559
14	ZINC000028115894	−	132.558
15	ZINC000044361247	Matairesinoside	132.365
16	ZINC000014780926	Lupalbigenin	132.295
17	ZINC000008662732	Prunetrin	132.26
18	ZINC000001577210	−	131.37
19	ZINC000004098459	Tremulacin	131.202
20	ZINC000028541553	Vincoside Lactam	131.008
21	vorasidenib	−	96.0148
22	ivosidenib	−	112.117

### ADME and toxicity prediction

To predict Pharmacological possessions of all the selected ligands and reference compounds, ADME module was conducted (Table 2). 8 compounds showed good aqueous solubility (water, 25°C) according to the aqueous solubility prediction. 19 compounds are non-inhibitors of CYP2D6, and 19 compounds had high or very high BBB level, which had better brain-blood barrier penetrating ability than Vorasidenib. Aimed at hepatotoxicity, 11 compounds were found to be safe which were less harmful than Vorasidenib and Ivosidenib. Results showed that 11 compounds were have a great human-intestinal absorption level. Besides, 5 compounds were good at being absorbed according to plasma protein binding properties. In this study, the issue of safety is also thoroughly examined. To verify the safety of the compounds chosen, the TOPKAT module was performed (Table 3). There are 12 compounds were nonmutagenic and 8 compounds had no developmental toxicity. However, Vorasidenib was identified to have high developmental toxicity potential.

**Table 2:** Adsorption, distribution, metabolism, and excretion properties of compounds.

**Number**	**Compounds**	**Solubility level**	**BBB level**	**CYP2D6**	**Hepatotoxicity**	**Absorption level**	**PPB level**
1	ZINC000004098643	2	4	0	0	2	1
2	ZINC000042851784	0	4	0	1	2	0
3	ZINC000049872065	3	4	0	0	2	0
4	ZINC000002033589	2	4	1	0	3	0
5	ZINC000038148193	0	4	0	1	2	0
6	ZINC000030726940	0	4	0	1	3	0
7	ZINC000028539727	4	4	0	1	3	0
8	ZINC000021992902	3	4	0	0	1	0
9	ZINC000049872393	3	4	0	0	3	0
10	ZINC000030729923	0	4	0	0	3	1
11	ZINC000008220036	0	4	0	0	3	1
12	ZINC000030726863	0	4	0	1	3	0
13	ZINC000014727602	2	4	0	1	1	0
14	ZINC000028115894	0	4	0	1	3	0
15	ZINC000044361247	3	4	0	0	3	0
16	ZINC000014780926	2	4	0	1	0	1
17	ZINC000008662732	3	4	0	1	3	0
18	ZINC000001577210	2	1	0	0	0	1
19	ZINC000004098459	3	4	0	0	3	0
20	ZINC000028541553	3	4	0	0	2	0
21	vorasidenib	1	1	0	1	0	1
22	ivosidenib	2	4	0	1	0	1

Abbreviations: BBB: blood-brain barrier; CYP2D6: cytochrome P-450 2D6; PPB: plasma protein binding. Aqueous-solubility level: 0, extremely low; 1, very low, but possible; 2, low; 3, good. BBB level: 0, very high penetrant; 1, high; 2, medium; 3, low; 4, undefined. CYP2D6 level: 0, noninhibitor; 1, inhibitor. Hepatotoxicity: 0, nontoxic; 1, toxic. Human-intestinal absorption level: 0, good; 1, moderate; 2, poor; 3, very poor. PPB: 0, absorbent weak; 1, absorbent strong.

**Table 3:** Toxicities of compounds.

**Number**	**Compounds**	**Mouse NTP**		**Rat NTP**		**Ames**	**DTP**
		**Female**	**Male**	**Female**	**Male**		
1	ZINC000004098643	0.997	0	1	0	0	0.997
2	ZINC000042851784	0	0	0	1	0.089	0
3	ZINC000049872065	0.353	0	0.752	0.006	0	1
4	ZINC000002033589	0	1	1	0.05	0.265	0.998
5	ZINC000038148193	0	0	0	1	0.08	0.411
6	ZINC000030726940	0	0	0.053	1	0.983	1
7	ZINC000028539727	1	1	1	0.019	0.998	0
8	ZINC000021992902	0.198	0	0.033	0.251	0	1
9	ZINC000049872393	0.204	0	1	0.009	0.992	0
10	ZINC000030729923	0.999	1	1	0	1	0
11	ZINC000008220036	0	1	1	0	1	0.998
12	ZINC000030726863	0	0	1	1	0.165	1
13	ZINC000014727602	0	1	1	1	0	0.501
14	ZINC000028115894	0	0	0.062	1	0.982	1
15	ZINC000044361247	0.856	0	1	0.008	0.991	1
16	ZINC000014780926	0	1	1	1	0	1
17	ZINC000008662732	0.996	1	0.672	1	0.478	0.04
18	ZINC000001577210	0	0.173	0	0.952	0	1
19	ZINC000004098459	0.005	0	0.988	0.003	0	0
20	ZINC000028541553	1	0	0.99	0	0.857	0.994
21	vorasidenib	0	1	0	0	0	0
22	ivosidenib	1	1	0	0	0	1

Abbreviations: NTP: U.S. National Toxicology Program; DTP: developmental toxicity potential. NTP < 0.3 (noncarcinogen); >0.8 (carcinogen). Ames < 0.3 (nonmutagen); >0.8 (mutagen). DTP < 0.3 (nontoxic); >0.8 (toxic).

Taking all the above results into account, ZINC000049872393 and ZINC000004098459 were considered to be valuable leading compounds with good solubility and absorption levels. They didn’t inhibit CYP2D6 and had no hepatotoxicity. Furthermore, compared with other compounds, they were anticipated to have lower rodent carcinogenicity, Ames mutagenicity, and developmental toxicity potential, which also strongly suggested their perspective application in drug development.

### Analysis of ligand binding and pharmacophore prediction

**Figure 2:**
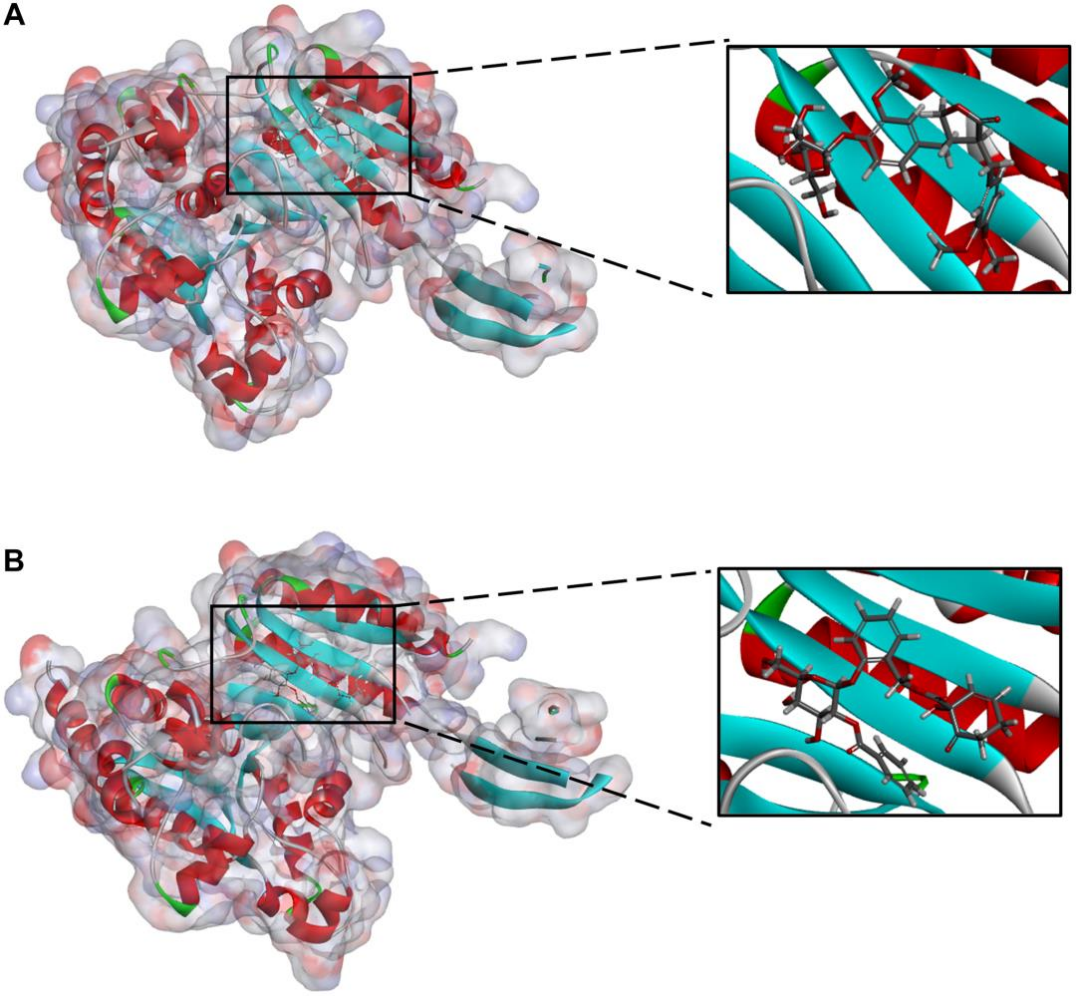
(**A**) ZINC000004098459-IDH1_R132H complex (**B**) ZINC000049872393 -IDH1_R132H complex.

**Figure 3:**
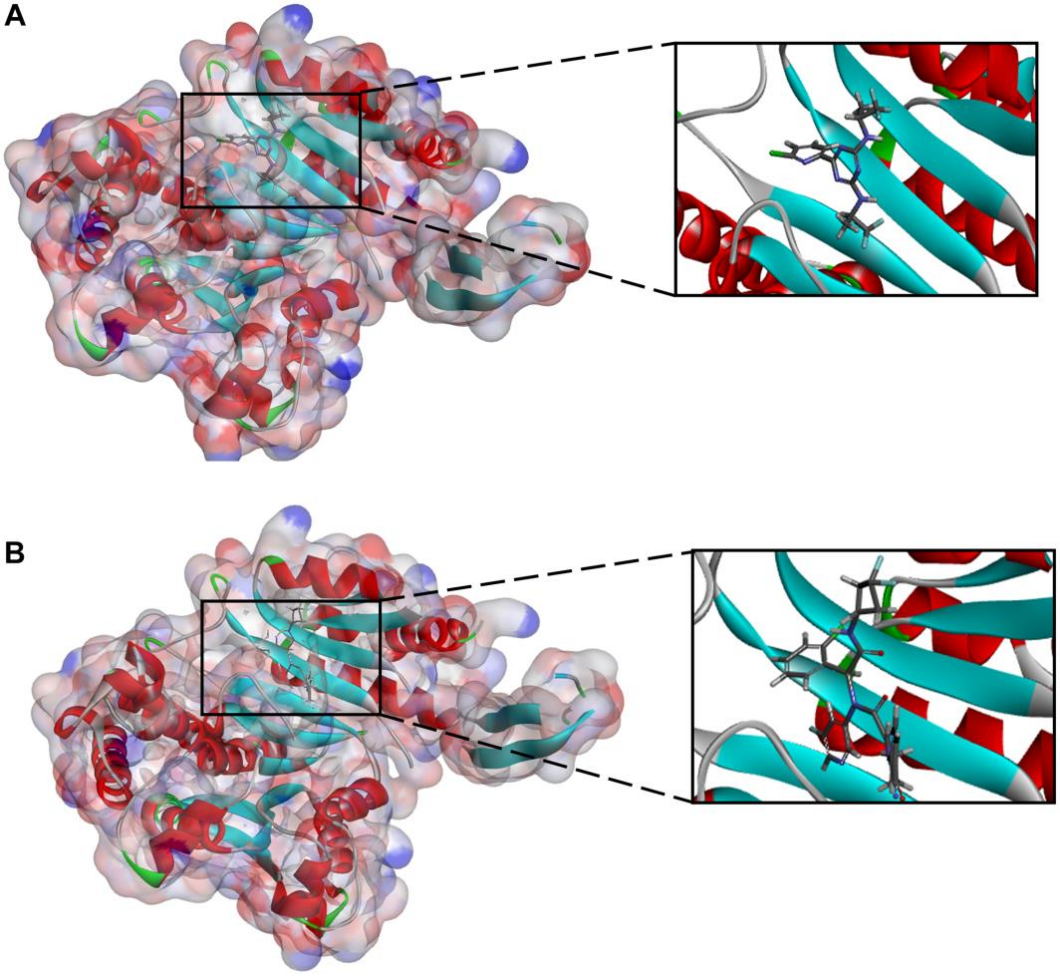
(**A**) Vorasidenib -IDH1_R132H complex (**B**) Ivosidenib-IDH1_R132H complex.

To evaluate ligand-binding mechanisms of ZINC000049872393, ZINC000004098459 and the reference compounds, the CDOCKER module were practiced to dock ZINC000049872393 and ZINC000004098459 into the protein structure of IDH1_R132H, and the potential energy was exhibited as listed in Table 4. The binding affinity according to CDOCKER point that ZINC000049872393 and ZINC000004098459 were stable binding with IDH1_R132H (Figures 2 and 3, and Supplementary Figure 1).

**Table 4:** CDOCKER potential energy of compounds with IDH1_RI32H.

**Compounds**	**-CDOCKER Potential Energy (kcal/mol)**
ZINC000049872393	50.2918
ZINC000004098459	55.8921
vorasidenib	35.0207
ivosidenib	36.3305

**Figure 4:**
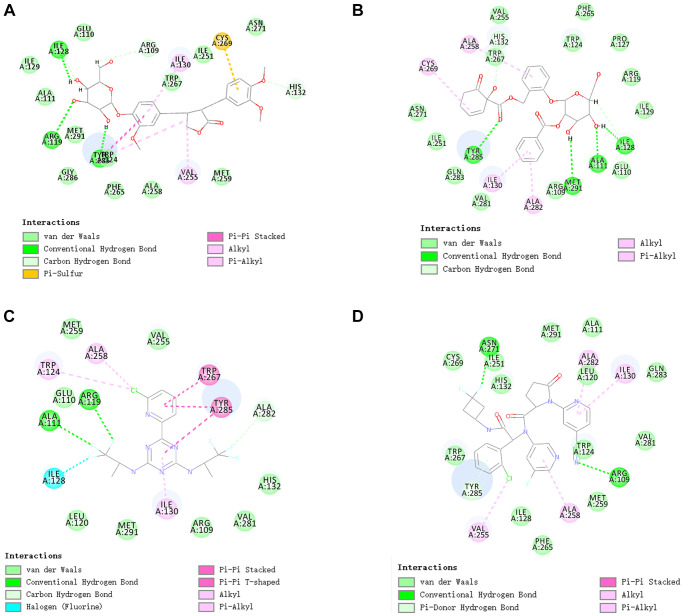
The inter-molecular interaction of the predicted binding modes of (**A**) ZINC000004098459 to IDH1_R132H; (**B**) ZINC000049872393 to IDH1_R132H (**C**) Vorasidenib to IDH1_R132H (**D**) Ivosidenib to IDH1_R132H.

**Figure 5:**
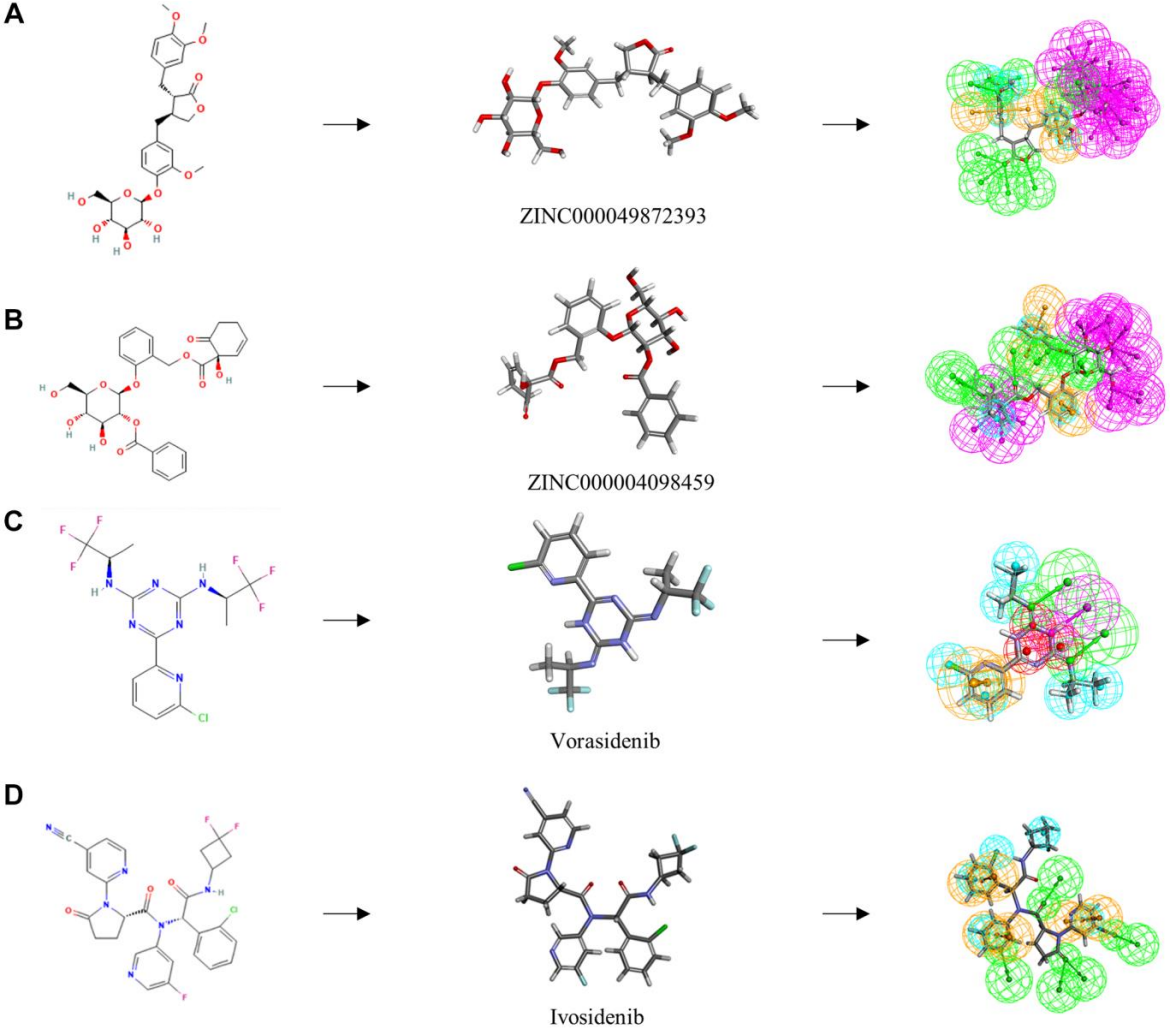
**The 2D structures of the reference compounds and novel compounds selected from virtual screening by chemdraw.** 3D structures of the reference compounds and novel compounds selected from virtual screening by DS 4.5. And Pharmacophore predictions using 3D-QSAR (Green represents hydrogen acceptor, and blue represents hydrophobic center and purple represents hydrogen donor). (**A**) ZINC000004098459 (**B**) ZINC000049872393 (**C**) Vorasidenib (**D**) Ivosidenib.

We also performed hydrogen and pi bonds interactions through structural computation study (Figure 4. Results pointed that ZINC000049872393 have 3 pairs of hydrogen bonds bind to IDH1_R132H, by O28 of the compound with A:ARG119:HH11 of IDH1_R132H, H60 of the compound with A:ILE128:O of IDH1_R132H, H64 of the compound with A:TYR285:O of IDH1_R132H. ZINC000004098459 formed 4 pairs of hydrogen bonds with IDH1_R132H, by A:ALA111:HN of IDH1_R132H with O8 of the compound, A:TYR285:HH of IDH1_R132H with O24 of the compound, A:MET291:SD of IDH1_R132H with H41 of the compound, H43 of the compound with A:ILE128:O of IDH1_R132H etc. Also, pi bonds interactions were listed in the complex. Both ZINC000049872393 and ZINC000004098459 formed 4 pairs of pi bonds bind to IDH1_R132H. what’s more, Vorasidenib and Ivosidenib formed 2 and 3 pairs of hydrogen bonds, 5 and 6 pairs of pi bonds interactions respectively (Tables 5 and 6). ZINC000004098459, ZINC000049872393 and the reference compounds displayed several feature pharmacophores, which showed 47 and 57 feature pharmacophores in ZINC000004098459 and ZINC000049872393, 12 and 16 feature pharmacophores in Vorasidenib and Ivosidenib (Figure 5).

**Table 5:** Hydrogen bond interaction parameters for each compound with IDH1_RI23H.

**Receptor**	**Compound**	**Donor atom**	**Receptor atom**	**Distances (Å)**
5l58 (IDH1_R132H)	ZINC000049872393	A:ARG119:HH11	ZINC000049872393:O28	2.88
		ZINC000049872393:H60	A:ILE128:O	1.97
		ZINC000049872393:H64	A:TYR285:O	1.95
	ZINC000004098459	A:ALA111:HN	ZINC000004098459:O8	1.95
		A:TYR285:HH	ZINC000004098459:O24	2.22
		ZINC000004098459:H41	A:MET291:SD	2.65
		ZINC000004098459:H43	A:ILE128:O	1.92
	vorasidenib	A:ALA111:HN	Molecule:F6	2.80
		A:ARG119:HH11	Molecule:F7	2.57
	ivosidenib	A:ARG109:HH11	Molecule:N13	2.75
		A:ASN271:HD22	Molecule:F3	2.12
		A:TYR285:HH	Molecule	1.91

**Table 6:** π-Related interaction parameters for each compound with IDH1_R132H.

**Receptor**	**Compound**	**Donor atom**	**Receptor atom**	**Distances (Å)**
5l58 (IDH1_R132H)	ZINC000049872393	A:TYR285	ZINC000049872393	4.82101
		ZINC000049872393	A:VAL255	4.52069
		A:TYR285	ZINC000049872393	5.39783
		ZINC000049872393	A:ILE130	5.21844
	ZINC000004098459	A:CYS269	ZINC000004098459	5.21158
		ZINC000004098459	A:ALA258	5.44202
		ZINC000004098459	A:ILE130	5.2495
		ZINC000004098459	A:ALA282	4.69611
	vorasidenib	A:TYR285	Molecule	4.18707
		A:TYR285	Molecule	5.02173
		A:TRP267	Molecule	5.21984
		A:ALA258	Molecule:Cl1	4.2239
		A:TRP124	Molecule:Cl1	5.08689
	ivosidenib	Molecule	A:ILE130	4.83303
		A:TYR285	Molecule	4.81399
		Molecule:Cl1	A:VAL255	3.87025
		Molecule	A:ILE130	5.25461
		Molecule	A:ALA282	4.32379
		Molecule	A:ALA258	5.36888

### Molecular dynamics simulation analysis

**Figure 6:**
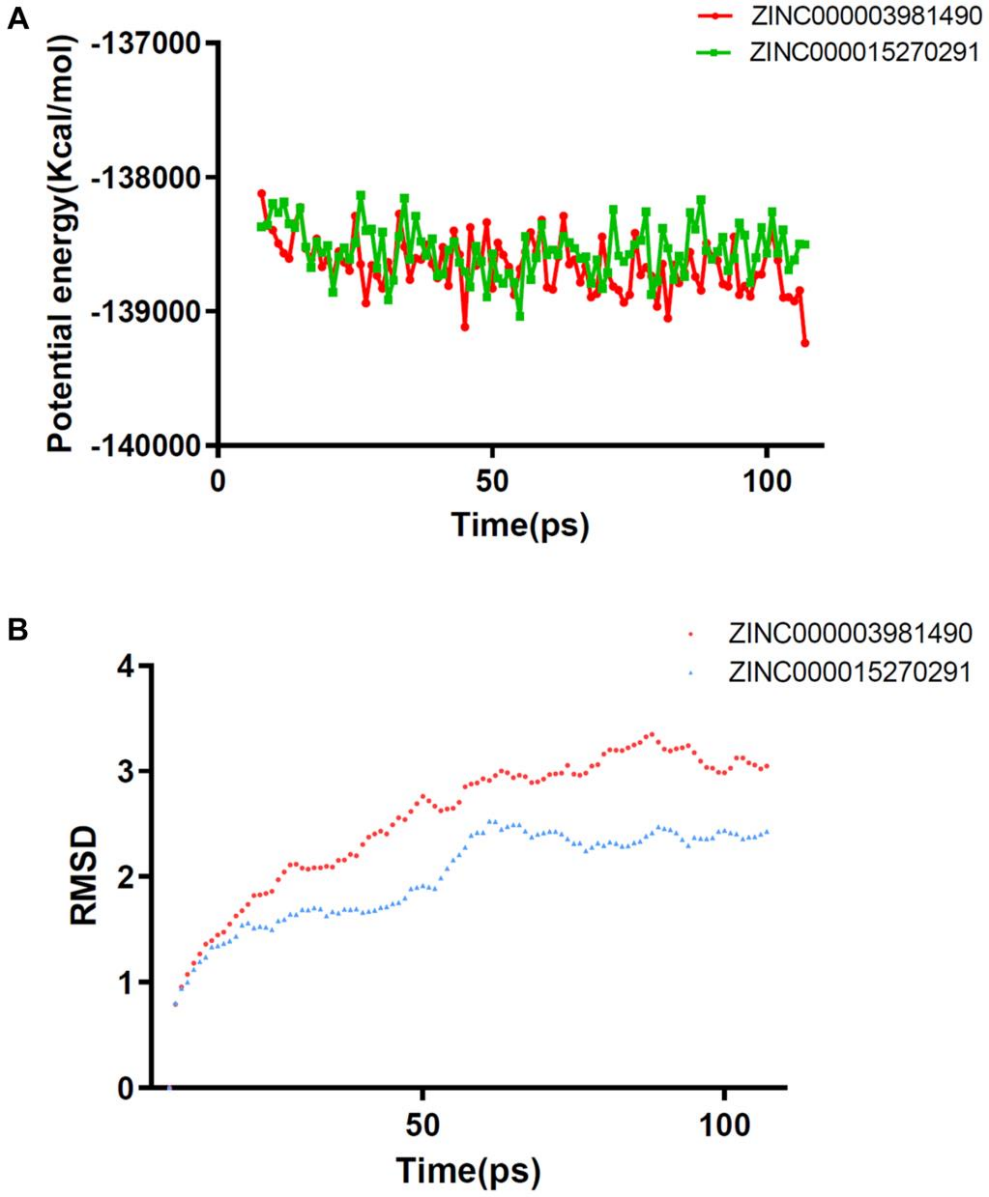
Results of molecular dynamics simulation of two complexes (**A**) Potential Energy (**B**) Average backbone RMSD.

Molecular dynamics simulation module was performed to value the stability of the ligand-IDH1 R132H complexes. Figure 6 depicted the RMSD curves and the potential energy chart for each complex. The complexes’ trajectories reached equilibrium, the RMSD and potential energy of each complex remained stable over time. The results verified that hydrogen bonds and pi bonds interaction formed by the compound and IDH1_R132H contribute to the steadiness of the complex as well. The final results show that these two compounds can not only interact with IDH1_R132H, but these complexes can also be stable at natural circumstances.

## DISCUSSION

Cholangiocarcinoma is hepatobiliary malignant cancer. The prevention and therapy of cholangiocarcinoma has attracted great attention in recent years [24]. Although novel approaches have brought hope to significantly improve the survival rate of cholangiocarcinoma patients, the patients’ prognosis is still poor. Mutations in IDH1_R132H induce conserved residues and stimulate neomorphic enzymatic function, leading to the accumulation of 2-hydroxyglutarate, which influences the formation of CpG methylation, resulting in aberrant gene expression and abnormal cell proliferation and differentiation, and abnormal gene expression [14, 25, 26]. In recent years, some academic researchers have developed new IDH1_R132H inhibitors that can be used in clinical applications, and combined therapy with radiotherapy and surgical treatment has turned out to be a research hotspot in the anti-tumor drugs field [27]. Compounds have made breakthroughs in the design and improvement of the IDH1_R132H inhibitor drug [28, 29]. However, due to various reasons, the current inhibitors have many limitations. Among these inhibitors, Ivosidenib is undoubtedly the most “developed” IDH inhibitor in CCA, with fatigue, nausea, diarrhoea, abdominal discomfort, decreased appetite, and vomiting being the most often reported adverse effects [30]. The top priority of this study is to identify and screen novel IDH1-R131H targeting drugs.

In this study, there are 17,931 molecules were downloaded for virtual screening. According to the LibDock score, we obtained the degree of energy majorization and conformational stability of the compound. Higher Libdock scores signified better energy majorization and conformation of the compound. Calculated by this module, 8763 nature products can stably bind to IDH1_R132H. The results indicate that these compounds appear to provide a more stable conformation and optimize energy structure compared with the reference compounds. For further research, the top 20 natural compounds were selected.

The ADME and TOPKAT predictions were performed to expose the pharmacological properties of the 20 compounds with top LibDock scores. Results showed that ZINC000049872393 and ZINC000004098459 were relatively high-quality drug candidates because they were soluble in water, have no hepatotoxicity, and have a good level of intestinal absorption, as well as non-inhibitors of CYP2D6. Moreover, low Ames mutagenicity and developmental toxicity potential were predicted. Therefore, ZINC000049872393 and ZINC000004098459 were identified as ideal candidate. These findings clarified their potential applications and future prospects in the realm of medication development. The rest of the drugs on the list can also be used for drug development by changing particular groups and atoms to lessen toxicity and negative effects. Taking the results of all the above reports into account, ZINC000049872393 and ZINC000004098459 should be performed in further analysis.

We also investigated the ligand-binding mechanism chemical bonds of ZINC000049872393 and ZINC000004098459 with IDH1_R132H. The calculation results of CDOCKER module computation explain the reason that the binding affinity of ZINC000049872393 and ZINC000004098459 with IDH1_R132H is significantly solid and stable. Finally, we evaluate the ligand-IDH1_R132H complexes in the natural environment by molecular dynamics simulations, and RMSD and potential energy were calculated. The results showed that the complexes' trajectories can reached equilibrium and were stable in the natural environment. To sum up, drug development can be prospectively conducted, such as modifications and refinements to make ligands and receptors more strongly bound.

This study is based on the screening of ideal lead compounds that can be used to elucidate the key steps in current drug design. The molecular conformation, pharmacological properties, binding affinity, and stability of each selected nature products were revealed using a comprehensive computational study. We concluded that the ZINC000049872393 and ZINC000004098459 may have the most potential in the current drug treatment of cholangiocarcinoma and glioblastoma. However, the candidate drugs still need to undergo thousands of refinements before they can be marketed. In addition, our study can be used as a guide to screen compounds that could have potential impacts. Only through such high-tech means can we screen out more lead compounds and thus improve current drug development.

In this study, a sequence of computer-aided structure and chemical techniques were conducted to screen ideal leading compounds that have the potential to inhibit the function of IDH1_R132H. ZINC000049872393 and ZINC000004098459 were selected as two important safety candidate drugs in the development of IDH1_R132H inhibitors. These two compounds provide a solid and reliable basis for IDH1_R132H targeted drugs design and development. In addition, the research also provided lots drug candidate, which can be used as a reliable reference for IDH1_R132H or other proteins in drug design and development. In conclusion, the goal of this study was to build a database of natural substances to uncover more possible therapeutic candidates that can block IDH mutations. Despite the fact that this study was carried out with a meticulous design and exact measurements, we must recognize that it had some limitations. No medicine may be marketed unless it is enhanced and refined. Several groups and atoms that can affect the pharmacological properties of the medications must be modified in order for these two compounds to be more suitable as drug candidates. More pharmaceutical safety indicators, such as MTD (Maximum Tolerated Dosage) and AB (Aerobic Biodegradability), should be researched in the future to support our findings. These restrictions will be the subject of our future research.

## MATERIALS AND METHODS

### Software and compounds library

Discovery Studio 4.5 software allows us to screen molecules based on their structure. It can work through chemical structure and biological calculations after simulating the molecular structure. The molecules that passed the initial screening can then be chemically changed to improve their potential as targeted medicines. This study makes extensive use of several Discovery Studio 4.5 modules. For initial filtering, the LibDock module is utilized. The ADME module is used to assess pharmacological features. To analyze molecular docking, the CDOCKER module was employed. The molecules we examined in this investigation were natural products from the ZINC15 database.

### Virtual screening by using LibDock

The Ligand-binding pocket region of IDH1_R132H was chosen as the binding site, and then virtual screening was performed [31–33]. Libdock is a rigid docking module when binds with a micromolecular ligand, which tends to have a small impact on the receptor. Download the 2.0-Å crystal structure of human IDH1_R132H, importing into the working environment of LibDock. After removing crystal water and other heteroatoms from the protein, hydrogen was added, followed by protonation, ionisation, and energy minimization [34, 35]. Following that, the binding site was defined using the manufactured protein, which was also chosen as the best docking site. Finally, all ligands were docked into this binding site, and the LibDock program was used to filter them realistically. Then, using the LibDock score, rank all docked poses.

### Absorption, distribution, metabolism, and excretion and toxicity prediction

A medication’s absorption, distribution, metabolism, and excretion are all significant pharmacological features. The ADME module is designed to predict the pharmacological features of drug candidates. Furthermore, the toxicity of medication candidates is critical. The TOPKAT (Toxicity Predicted by Komputer Assisted Technology) module was used to predict the toxicity of drug candidates, including Ames mutagenicity, rodent carcinogenicity, hepatotoxicity, and developmental toxicity potential. The physicochemical properties of IDH1_R132H drug candidates should be properly studied and forecasted while designing these medications.

### Molecule docking and pharmacophore prediction

The CDOCKER module, which is based on the CHARMm36 Force Field, was used to analyze molecular docking and can yield reliable molecular docking results. During docking, the receptor remains rigid, but the ligand can be bent. Each complex attitude’s CHARMm energy (interaction energy plus ligand strain) and interaction energy were then calculated and analysed. These findings aided in determining ligand binding affinity. Then, IDH1_R132H’s crystal structure was obtained from the protein database. Furthermore, we eliminated the semi-flexible and rigid docking of crystal molecules to water, which may have an impact on receptor-ligand complex formation. Furthermore, water molecules were eliminated. Meanwhile, we supplied the protein with hydrogen atoms. The ligand was then removed from the IDH1_R132H binding site and re-docked, and the binding’s reliability was validated by comparing the root mean square deviation of the two conformations. The IDH1_R132H binding site was determined as a region with a radius of 16 Å around the geometric centre of IDH1_R132H.Following that, the ligand was placed into IDH1_R132H’s binding pocket, and the CDOCKER module was run. Finally, we used 3D-QSAR module to construct and display the pharmacophores of the molecules.

### Molecular dynamics simulation

The optimum ligand-IDH1_R132H conformation is supplied into the molecular mechanics simulation module after the molecular insertion procedure is completed. A water model with explicit periodic boundary solvation was used to solve the ligand-IDH1_R132H complex in an orthorhombic box. After that, sodium chloride with an ionic strength of 0.145 was added to the system to mimic the physical environment. After that, the CHARMM field is simulated. The ligand was parameterized using the CHARMM field. After that, the ligand was put through 1000 energy minimization steps, 500 of which were steepest descent and 500 of which were conjugate gradient minimization. Finally, 0.227 is the root mean square deviation. The system temperature was slowly increased from 50 K to 300. Finally, using the initial complicated arrangement as a guide, the trajectory is generated using root mean square deviation (RMSD), potential energy, and structural parameters, and the trajectory protocol is tested in Discovery Studio 4.5.

## Supplementary Materials

Supplementary Figure 1
