# Supplementary material for: Computational study on novel natural compound inhibitor targeting IDH1_R132H
Source: Aging (Albany NY). 2022 Jul 7;14(13):5478–92. doi: 10.18632/aging.204162 (PMC9320544; doi:10.18632/aging.204162)
Supplement: Supplementary Figure 1 [file aging-14-204162-s001.pdf]

SUPPLEMENTARY FIGURE

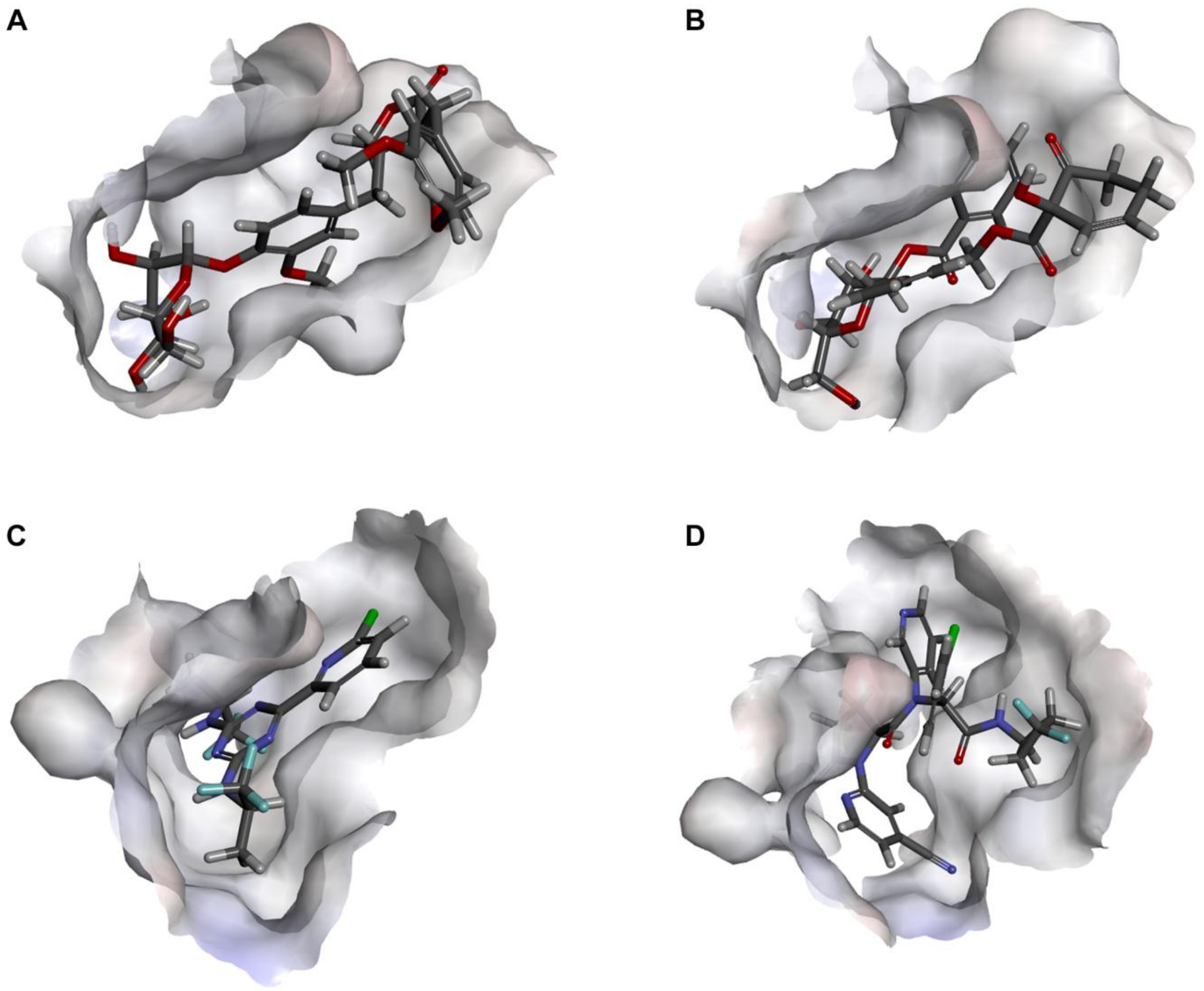

Supplementary Figure 1. Schematic drawing of interactions between ligands and IDH1_R132H and the Ionizability surface of the junction pocket was added, blue represented basic ionization, red represented acid ionization, and ligands were shown in sticks, the structure around the ligand-receptor junction were shown in thinner sticks. (**A**) ZINC000004098459 (**B**) ZINC000049872393 (**C**) Vorasidenib (**D**) Ivosidenib.
